# Stability Criteria for Nonlinear-Truncated V-Fractional-Order Derivative Systems with Applications to Synchronization

**DOI:** 10.3390/e28040399

**Published:** 2026-04-01

**Authors:** Wengui Yang

**Affiliations:** 1 School of Education and Humanities, Sanmenxia Polytechnic, Sanmenxia 472000, China; yangwengui@smxpt.edu.cn; 2 College of Application of Engineering, Henan University of Science and Technology, Sanmenxia 472000, China

**Keywords:** truncated V-fractional-order derivative systems, global exponential stability, global asymptotic stability, modified projective synchronization, Bellman–Gronwall inequality, 93D20, 93B52, 37D45, 26A33

## Abstract

This paper investigates the stability of nonlinear systems with truncated V-fractional-order derivatives. Initially, based on the fundamental properties of V-fractional calculus, the Bellman–Gronwall inequality for V-fractional α-differentiable functions is derived. Subsequently, several sufficient conditions for the stability of the considered systems are established via the Lyapunov direct method. For practical applications, multiple synchronization criteria for drive-response systems are further deduced by leveraging the aforementioned stability results. Finally, numerical examples are presented to verify the effectiveness and feasibility of the main theoretical findings.

## 1. Introduction

Fractional calculus, as a natural generalization of classical integer-order calculus, traces its origins back to the 17th century through a celebrated correspondence between Leibniz and L’Hôpital on the meaning of the half-order derivative [1]. Despite its long theoretical history, fractional calculus remained a purely theoretical pursuit for centuries with limited practical applications. However, over the past three to four decades, driven by the growing demand for accurate modeling of complex dynamic systems in engineering, physics, biology, chemistry, and economics, fractional calculus has witnessed a remarkable renaissance and emerged as a powerful mathematical tool for describing intricate real-world phenomena [2,3]. Unlike integer-order calculus, which only captures local and instantaneous dynamic behaviors, fractional calculus inherently exhibits non-locality and memory effects. These unique properties enable it to effectively characterize systems with historical dependence and hereditary attributes, such as viscoelastic materials [4], biological neural networks with long-term synaptic plasticity [5], electrochemical processes with slow diffusion dynamics [6], and financial markets influenced by historical economic events [7].

Nonlinear fractional-order systems integrate the non-local memory characteristics of fractional calculus with the inherent complexity of nonlinear dynamics, forming a class of dynamic systems with more sophisticated and diverse behaviors than their integer-order counterparts. Hence, nonlinear fractional-order systems have attracted considerable attention in recent decades due to their inherent ability to describe the memory and hereditary properties of complex nonlinear systems in nature and engineering. For instance, nonlinear fractional-order systems can exhibit rich dynamic phenomena such as fractional-order chaos (e.g., Lorenz [8,9,10], Chua [11,12], and Chen systems [13,14]), multi-stability (i.e., the coexistence of multiple equilibrium states or periodic orbits) [15,16], and power-law decay/growth responses [17,18]—behaviors that are difficult to replicate using integer-order models. These unique dynamic characteristics make nonlinear fractional-order systems particularly suitable for modeling and analyzing complex real-world systems, but they also present substantial challenges for their theoretical analysis and practical control.

Stability is one of the most fundamental and critical properties of dynamic systems, serving as a cornerstone for assessing system performance, designing control schemes, and guaranteeing reliable operational behavior. For nonlinear fractional-order systems, stability analysis is not only a core theoretical pillar in fractional-order system theory but also a prerequisite for their successful deployment across diverse engineering and scientific fields [19,20,21]. In practical applications, the stability of nonlinear fractional-order systems directly dictates the safety and efficacy of the system. For example, in uncertain fractional-order systems, the stability of the closed-loop system ensures stable operation and mitigates the risk of equipment damage [22,23,24,25,26]. In biomedical engineering, the stability of fractional-order models for physiological systems is pivotal for disease diagnosis and the design of therapeutic devices [27]. In chaotic secure communication, the stability of the synchronization error system between the transmitter (drive system) and receiver (response system) is the linchpin for achieving reliable information transmission [28]. Additionally, in robotics and autonomous vehicles, fractional-order controllers are increasingly employed for their superior performance, and the stability analysis of nonlinear fractional-order systems is indispensable for ensuring motion precision and operational safety [29].

Over the past few decades, researchers worldwide have carried out extensive and in-depth investigations on the stability analysis of nonlinear fractional-order systems based on Riemann–Liouville, Caputo, Hadamard, and Atangana–Baleanu fractional calculus, yielding a vast body of theoretical results and analytical methodologies [30,31,32,33]. For instance, Wen et al. [34] proved the stability theorem for nonlinear fractional-order differential equations using the Gronwall–Bellman lemma, designed a linear state feedback controller (with parameters tuned via pole placement), and verified its efficacy through numerical simulations of the fractional-order Lorenz system. Yu et al. [35] investigated the generalized Mittag–Leffler stability of multivariable fractional-order nonlinear systems using a fractional Lyapunov direct method. Under different conditions, Yuan et al. [36] addressed the mean-square asymptotic stability of fractional-order nonlinear stochastic systems by directly employing the properties of integral solutions and the Mittag–Leffler function. However, owing to the inherent complexity of traditional fractional calculus—including non-locality, memory effects, and the non-commutativity of fractional operators—as well as the diversity of nonlinear terms, the stability analysis of these systems remains far more challenging than that of integer-order nonlinear systems. Many classical stability theories and methods developed for integer-order systems, such as Lyapunov stability criteria and frequency–domain methods, cannot be directly generalized to nonlinear fractional-order systems. This has spurred the development of novel theoretical frameworks, analytical tools, and stability criteria specifically tailored to nonlinear fractional-order systems.

However, in 2017, Sousa and Oliveira [37] first introduced the truncated V-fractional calculus, which not only subsumes several existing fractional-order calculus formulations (see Remark 3) but also inherits the desirable properties of integer-order calculus. For example, Souahi et al. [38] studied the stability properties (including asymptotic stability) of conformable fractional-order nonlinear systems by means of the Lyapunov direct method, where the corresponding conformable fractional derivative is governed by a single parameter only. Using the constructed conformable Adomian decomposition method, He et al. [39] derived numerical solutions for conformable fractional-order linear and nonlinear equations. Based on linear matrix inequalities, Mayo-Maldonado et al. [40] addressed the stability of linear conformable fractional-order differential systems. Via the Halanay inequality with average impulsive intervals, Luo et al. [41] studied the fractional exponential stability of nonlinear conformable fractional-order delayed systems and the fractional exponential synchronization of conformable fractional-order delayed inertial neural networks with delayed impulses. Using the improved modified extended tanh-function method, Bossly et al. [42] conducted a comprehensive linear stability analysis to examine the stability of solutions for the truncated M-fractional fifth-order Korteweg–de Vries equation. The fractional-order calculus frameworks employed in prior studies can be regarded as special cases of the truncated V-fractional calculus.

Although extensive research has been devoted to the stability analysis of fractional-order differential systems, the stability and synchronization of nonlinear truncated V-fractional-order derivative systems remain largely unexplored. This paper aims to fill this research gap by investigating the stability and synchronization of such systems. Specifically, we consider the following nonlinear truncated V-fractional-order derivative system:

(1) Vγ,β,αδ,p,qiρy(t)=ψ(t,y(t)),t>t0≥0,y(t0)=y0,

where $y\in R^{n}$, $\psi :R_{+}\times R^{n}\to R^{n}$ is a given nonlinear function satisfying ψ(t,0)=0, and Vγ,β,αδ,p,qiρ denotes the truncated V-fractional derivative of order 0<α<1 satisfying Definition 3. The main contributions of this paper can be summarized as follows.

- To the best of the authors’ knowledge, this work constitutes the first investigation into the stability analysis of nonlinear systems with the truncated V-fractional-order derivative, which is governed by six distinct parameters.
- A generalized Bellman–Gronwall inequality for V-fractional α-differentiable functions is established, which subsumes various Bellman–Gronwall-type inequalities by tuning different parameters within the V-fractional calculus framework.
- Novel sufficient conditions for the V-fractional global exponential stability and global asymptotic stability of the considered systems are derived using the Lyapunov direct method; these conditions reduce to the stability results for conformable fractional-order nonlinear systems reported in [38].
- New modified projective synchronization criteria for drive-response systems are deduced by leveraging the established stability theoretical results. Novel numerical schemes are proposed to predict the chaotic dynamics of Rucklidge and Shimizu–Morioka systems involving the truncated *M*-fractional derivative [18]. Furthermore, the truncated V-fractional-order derivative mentioned in this work can be adopted to generalize the corresponding chaotic behaviors.
- Practical application examples are provided to validate the effectiveness and feasibility of the proposed stability and synchronization theories. The exponential stability of some neural networks with conformable fractional derivative was investigated [41,43]. Furthermore, the theoretical results derived in this paper are also applicable to the investigation of stability and synchronization for the relevant truncated V-fractional derivative neural networks.

The remainder of this paper is structured as follows. Section 2 introduces the basic concepts and fundamental properties of truncated V-fractional calculus, as well as the stability definitions for the nonlinear truncated V-fractional-order derivative system (1); this section also presents the Bellman–Gronwall inequality for V-fractional α-differentiable functions. Section 3 derives several sufficient conditions for the stability of the considered systems using the Lyapunov direct method. Then, we apply the main stability results to the modified projective synchronization of drive-response systems in Section 4. Section 5 concludes the paper and outlines promising directions for future research in this field.

## 2. Preliminaries

In this section, we first introduce the six-parameter Mittag–Leffler function, which plays a crucial role in the development of this work.

**Definition 1**  (Six-parameter Mittag–Leffler function [44])**.** *Let γ,β,ρ,δ∈C and p,q>0 satisfy Re(γ)>0, Re(β)>0, Re(ρ)>0, Re(δ)>0, and Re(γ)+p≥q. The six-parameter Mittag–Leffler function is defined as*

(2) $$\begin{array}{c} E_{\gamma ,\beta ,p}^{\rho ,\delta ,q}\left(z\right)=\sum_{k=0}^{\infty }\frac{{\left(\rho \right)}_{qk}}{{\left(\delta \right)}_{pk}}\frac{z^{k}}{\Gamma (\gamma k+\beta )}, \end{array}$$

*where ${\left(\rho \right)}_{qk}$ denotes the generalized Pochhammer symbol, given by ${\left(\rho \right)}_{qk}=\Gamma \left(\rho +qk\right)/\Gamma \left(\rho \right)$.*

**Remark 1.**  *By setting p=δ=q=ρ=β=1, p=δ=q=ρ=1, p=δ=q=1, p=δ=1, and p=1 in *(2)* respectively, we obtain the one-, two-, three-, four- and five-parameter Mittag-Leffler functions [45,46,47,48,49]. When p=δ=q=ρ=β=γ=1, the function reduces to the exponential function $e^{z}$.*

**Definition 2**  (Six-parameter truncated Mittag–Leffler function [37])**.** *Let γ,β,ρ,δ∈C and p,q>0 satisfy Re(γ)>0, Re(β)>0, Re(ρ)>0, Re(δ)>0, and Re(γ)+p≥q. The six-parameter truncated Mittag-Leffler function is defined as*

(3) Eγ,β,pρ,δ,qi(z)=∑k=0i(ρ)qk(δ)pkzkΓ(γk+β).

**Remark 2.**  *By setting p=δ=q=ρ=β=1, p=δ=q=ρ=1, p=δ=q=1, p=δ=1, and p=1 in *(3)*, respectively, we obtain the one-, two-, three-, four- and five-parameter truncated Mittag–Leffler functions. Taking the limit i→∞ on both sides of *(3)* and choosing appropriate parameters, we can derive the one-, two-, three-, four-, five-, and six-parameter Mittag–Leffler functions, respectively. For p=δ=q=ρ=β=1 in *(3)*, applying the limits i→0, i→1 and i→∞ yields E0(z)=E1,1,11,1,10(z)=1, E1(z)=E1,1,11,1,11(z)=1+z, and E∞(z)=E1,1,11,1,1∞(z)=ez=exp(z), respectively.*

Based on the newly defined generalized truncated function in the six-parameter truncated Mittag–Leffler Function *(3)* and the gamma function Γ(β), Sousa and Oliveira [37] introduced the truncated V-fractional derivative.

**Definition 3**  (Truncated V-fractional-order derivative [37])**.** *For 0<α<1, let f:[0,∞)→R. We define the truncated V-fractional derivative of f of order α as*

(4) Vγ,β,αδ,p,qiρf(t)=limε→0ftHγ,β,pρ,δ,qi(εt−α)−f(t)ε,∀t>0,Vγ,β,αδ,p,qiρf(0)=limt→0+Vγ,β,αδ,p,qiρf(t),

*where Hγ,β,pρ,δ,qi(·) denotes the generalized truncated function defined by Hγ,β,pρ,δ,qi(z)=Γ(β)Eγ,β,pρ,δ,qi(z), and γ,β,ρ,δ∈C, p,q>0 satisfy Re(γ),Re(β),Re(ρ),Re(δ)>0 and Re(γ)+p≥q.*

**Remark 3.**  *If the α-th order truncated V-fractional derivative of a function f exists, we simply refer to f as V-fractional α-differentiable. By selecting different parameters in *(4)*, the truncated V-fractional derivative can be reduced to several well-known fractional derivatives, including the conformable fractional derivative, the alternative fractional derivative* (*and its truncated version*)*, and the M-fractional-order derivative* (*and its truncated variant*).

**Definition 4**  (V-fractional-order integral [37])**.** *For 0<α<1 and a>0, let f:[a,∞)→R. We define the truncated V-fractional-order integral of f of order α as*

(5) Iγ,β,αδ,p,qaρf(t)=Γ(γ+β)(δ)pΓ(β)(ρ)q∫atf(x)x1−αdx=ϖ∫atf(x)x1−αdx,∀t≥a,

*where $\varpi =\frac{\Gamma \left(\gamma +\beta \right){\left(\delta \right)}_{p}}{\Gamma \left(\beta \right){\left(\rho \right)}_{q}}$, γ,β,ρ,δ∈C and p,q>0 satisfy Re(γ), Re(β), Re(ρ), Re(δ)>0 and Re(γ)+p≥q, and ${\left(\rho \right)}_{q}$ denotes the Pochhammer symbol* (*rising factorial*) *defined as ${\left(\rho \right)}_{q}=\Gamma \left(\rho +q\right)/\Gamma \left(\rho \right)$.*

Next, we present some fundamental properties of the truncated V-fractional-order derivative and V-fractional integral.

**Lemma 1**  ([37])**.** *For 0<α≤1, let f:[0,∞)→R be V-fractional α-differentiable at $t_{0}>0$. Then f is continuous at $t_{0}$.* 

**Lemma 2**  ([37])**.** *Let 0<α≤1, γ,β,ρ,δ∈C and p,q>0 satisfy Re(γ),Re(β),Re(ρ),Re(δ)>0 and Re(γ)+p≥q, and let f,g be V-fractional α-differentiable functions. Then, for t>0, the following properties hold:*

*1.*  *Vγ,β,αδ,p,qiρ(af+bg)(t)=aVγ,β,αδ,p,qiρf(t)+bVγ,β,αδ,p,qiρg(t) for any a,b∈R;*
*2.*  *Vγ,β,αδ,p,qiρ(fg)(t)=f(t)Vγ,β,αδ,p,qiρg(t)+g(t)Vγ,β,αδ,p,qiρf(t);*
*3.*  *Vγ,β,αδ,p,qiρfg(t)=g(t)Vγ,β,αδ,p,qiρf(t)−f(t)Vγ,β,αδ,p,qiρg(t)[g(t)]2;*
*4.*  *Vγ,β,αδ,p,qiρ(c)=0, where c is a constant;*
*5.*  *If f is differentiable, then Vγ,β,αδ,p,qiρf(t)=t1−αϖdf(t)dt;*
*6.*  *(Chain rule) Vγ,β,αδ,p,qiρ(f∘g)(t)=f′(g(t))Vγ,β,αδ,p,qiρg(t), where f is differentiable at g(t).*

**Remark 4.** (i) *For 0<α≤1, let f:[0,∞)→R be V-fractional α-differentiable. It follows from Lemma 2 that $f^{2}:\left[0,\infty \right)\to R$ is also V-fractional α-differentiable, and Vγ,β,αδ,p,qiρf2(t)=2f(t)Vγ,β,αδ,p,qiρf(t) for all t∈[0,∞).*

(ii) *Let $f:\left[0,\infty \right)\to R^{n}$ be V-fractional α-differentiable. It follows from Lemma 2 that $f^{T}f:\left[0,\infty \right)\to R$ is also V-fractional α-differentiable, and Vγ,β,αδ,p,qiρ(fTf)(t)=2fT(t)Vγ,β,αδ,p,qiρf(t) for all t∈[0,∞).*

(iii) *Let $f:\left[0,\infty \right)\to R^{n}$ be V-fractional α-differentiable and P a symmetric positive definite matrix. It follows from Lemma 2 that $f^{T}Pf:\left[0,\infty \right)\to R$ is also V-fractional α-differentiable, and Vγ,β,αδ,p,qiρ(fTPf)(t)=2fT(t)PVγ,β,αδ,p,qiρf(t) for all t∈[0,∞).* 

**Lemma 3**  ([37])**.** *Let 0<α≤1, γ,β,ρ,δ∈C and p,q>0 satisfy Re(γ),Re(β),Re(ρ),Re(δ)>0 and Re(γ)+p≥q, and let f,g be V-fractional α-differentiable functions. Then, for t>0, the following identities hold:*

*1.*  *Vγ,β,αδ,p,qiρsintαα=1ϖcostαα, which implies Vγ,β,αδ,p,qiρsinϖtαα=cosϖtαα;*
*2.*  *Vγ,β,αδ,p,qiρcostαα=−1ϖsintαα, which implies Vγ,β,αδ,p,qiρcosϖtαα=−sinϖtαα;*
*3.*  *Vγ,β,αδ,p,qiρexptαα=1ϖexptαα, which implies Vγ,β,αδ,p,qiρexpϖtαα=expϖtαα.*

**Lemma 4**  (Lagrange mean value theorem for V-fractional α-differentiable functions [37])**.** *For a>0 and 0<α≤1, let f:[a,b]→R be continuous and V-fractional α-differentiable on (a,b). Then there exists ξ∈(a,b) such that*

(6) Vγ,β,αδ,p,qiρf(ξ)=f(b)−f(a)bαα−aαα,

*where γ,β,ρ,δ∈C and p,q>0 satisfy Re(γ),Re(β),Re(ρ),Re(δ)>0 and Re(γ)+p≥q.* 

**Lemma 5**  ([37])**.** *For a≥0, t≥a and 0<α≤1, let f be a continuous function such that Iγ,β,αδ,p,qaρf(t) exists, and let g:(a,b)→R be a V-fractional α-differentiable function. Then*

(7) Vγ,β,αδ,p,qiρIγ,β,αδ,p,qaρf(t)=f(t)andIγ,β,αδ,p,qaρVγ,β,αδ,p,qiρg(t)=g(t)−g(a),

*where γ,β,ρ,δ∈C and p,q>0 satisfy Re(γ),Re(β),Re(ρ),Re(δ)>0 and Re(γ)+p≥q.*

**Lemma 6.**  *For 0<α≤1, let f:[0,∞)→R be V-fractional α-differentiable. If Vγ,β,αδ,p,qiρf(t)≥0 for all t∈[0,∞), then f is increasing; if Vγ,β,αδ,p,qiρf(t)≤0 for all t∈[0,∞), then f is decreasing.*

**Proof.**  Let $t_{1},t_{2}\in \left[0,\infty \right)$ with $t_{1}<t_{2}$. It follows from Lemma 1 that $f:[t_{1},t_{2}]\to R$ is continuous and V-fractional α-differentiable on $\left(t_{1},t_{2}\right)$, which satisfies the conditions of the Lagrange mean value theorem for V-fractional α-differentiable functions. By Lemma 4, there exists $\xi \in (t_{1},t_{2})$ such that

(8) f(t2)−f(t1)=Vγ,β,αδ,p,qiρf(ξ)t2αα−t1αα.

Since $t_{1}<t_{2}$ and 0<α<1, we have $t_{2}^{\alpha }/\alpha -t_{1}^{\alpha }/\alpha >0$. If Vγ,β,αδ,p,qiρf(t)≥0 for all t∈[0,∞), then from (8) we obtain $f\left(t_{2}\right)\geq f\left(t_{1}\right)$, i.e., *f* is increasing. Similarly, if Vγ,β,αδ,p,qiρf(t)≤0 for all t∈[0,∞), then *f* is decreasing. This completes the proof of Lemma 6. □

**Lemma 7**  (Bellman–Gronwall inequality for V-fractional α-differentiable functions)**.** *For 0<α≤1 and $t_{0}\geq 0$, let $f:[t_{0},\infty )\to R$ be V-fractional α-differentiable and $g:[t_{0},\infty )\to R$ a continuous function such that Vγ,β,αδ,p,qiρf(t)≤g(t)f(t). Then the following inequality holds:*

(9) f(t)≤f(t0)expIγ,β,αδ,p,qt0ρg(t).

**Proof.**  Define h(t)=expIγ,β,αδ,p,qt0ρg(t). By Lemmas 2 and 3, we have

(10) Vγ,β,αδ,p,qiρh(t)=g(t)expIγ,β,αδ,p,qt0ρg(t)=g(t)h(t).

From the definition of h(t), we get $h(t_{0})=1$ and h(t)>0 for $t>t_{0}$. Using Lemma 2, (10) and the condition Vγ,β,αδ,p,qiρf(t)≤g(t)f(t), we derive that

(11) Vγ,β,αδ,p,qiρf(t)h(t)=h(t)Vγ,β,αδ,p,qiρf(t)−f(t)Vγ,β,αδ,p,qiρh(t)[h(t)]2≤h(t)g(t)f(t)−f(t)g(t)h(t)[h(t)]2=0.

Combining (11) with Lemma 6 implies that f(t)/h(t) is decreasing. Thus, $f\left(t\right)/h\left(t\right)\leq f\left(t_{0}\right)/h\left(t_{0}\right)=f\left(t_{0}\right)$, which yields f(t)≤f(t0)h(t)=f(t0)expIγ,β,αδ,p,qt0ρg(t). This completes the proof of Lemma 7. □

Setting g(t)=−λ in Lemma 7, we obtain the following corollary, whose proof is omitted.

**Corollary 1.**  *For 0<α≤1 and $t_{0}\geq 0$, let $f:[t_{0},\infty )\to R$ be V-fractional α-differentiable such that Vγ,β,αδ,p,qiρf(t)≤−λf(t), where λ>0 is a constant. Then $f\left(t\right)\leq f\left(t_{0}\right)\exp\left(-\lambda \varpi \frac{t^{\alpha }-t_{0}^{\alpha }}{\alpha }\right)$.*

**Definition 5**  (V-fractional exponential stability)**.** *The origin of system *(1)* is said to be V-fractionally exponentially stable if the inequality $∥y\left(t\right)∥\;\leq \;K∥y_{0}{∥}^{\nu }\exp\left(-\lambda \varpi \frac{t^{\alpha }-t_{0}^{\alpha }}{\alpha }\right)$ holds for all $t\geq t_{0}$, where ν,λ,K>0 are constants.*

**Definition 6.**  *For the origin of system *(1)*, we define the following stability concepts:*

*1.*  *It is called stable if, for all ε>0 and $t_{0}\in R_{+}$, there exists $\delta =\delta (\varepsilon ,t_{0})>0$ such that for any initial state $y_{0}\in R^{n}$ with $∥y_{0}∥\;<\;\delta$, we have ∥y(t)∥ < ε for all $t\geq t_{0}$.*
*2.*  *It is called attractive if, for any $t_{0}\geq 0$, there exists a positive constant $c=c(t_{0})$ such that for any $y_{0}\in R^{n}$ with $∥y_{0}∥\;<\;c$, we have $\lim_{t\to \infty }y\left(t\right)=0$.*
*3.*  *It is globally attractive if, for every initial condition $y_{0}\in R^{n}$, $\lim_{t\to \infty }y\left(t\right)=0$.*
*4.*  *It is asymptotically stable if it is both stable and attractive.*
*5.*  *It is globally asymptotically stable if it is stable and globally attractive.*

**Definition 7.**  *A continuous function $\vartheta :R_{+}\to R_{+}$ is said to be of class K if it is strictly increasing and satisfies ϑ(0)=0. We say ϑ is of class $K_{\infty }$ if $\lim_{s\to +\infty }\vartheta \left(s\right)=+\infty$.*

## 3. Main Results

In this section, based on the Lyapunov direct method, we derive several sufficient conditions for the stability of the considered system (1).

**Theorem 1.**  *Suppose y=0 is an equilibrium point of system *(1)* and $L:R_{+}\times R^{n}\to R$ is a continuous function. Furthermore, if the following conditions hold:*

*(A1)*  *$\varrho_{1}{∥y\left(t\right)∥}^{\mu }\leq L\left(t,y\left(t\right)\right)\leq \varrho_{2}{∥y\left(t\right)∥}^{\mu \nu }$ for some positive constants $\varrho_{1},\varrho_{2},\mu ,\nu$;*
*(A2)*  *L(t,y(t)) is V-fractional α-differentiable for all $t>t_{0}\geq 0$;*
*(A3)*  *Vγ,β,αδ,p,qiρL(t,y(t))≤−ϱ3∥y(t)∥μν for some positive constant $\varrho_{3}$.*

*Then the origin of system *(1)* is V-fractionally exponentially stable.*

**Proof.**  According to conditions (A1) and (A3), we derive

(12) Vγ,β,αδ,p,qiρL(t,y(t))≤−ϱ3ϱ2−1L(t,y(t)).

Applying Corollary 1 to inequality (12), we obtain

(13) $$\begin{array}{c} L\left(t,y\left(t\right)\right)\leq L\left(t_{0},y\left(t_{0}\right)\right)\exp\left(-\varrho_{3}\varrho_{2}^{-1}\varpi \frac{t^{\alpha }-t_{0}^{\alpha }}{\alpha }\right),\;\forall t\geq t_{0}. \end{array}$$

From condition (A1), the following inequalities hold:

(14) $$\begin{array}{cc} \varrho_{1}{∥y\left(t\right)∥}^{\mu } & \leq L\left(t,y\left(t\right)\right)\leq L\left(t_{0},y\left(t_{0}\right)\right)\exp\left(-\varrho_{3}\varrho_{2}^{-1}\varpi \frac{t^{\alpha }-t_{0}^{\alpha }}{\alpha }\right)\\ \; & \leq \varrho_{2}{∥y\left(t_{0}\right)∥}^{\mu \nu }\exp\left(-\varrho_{3}\varrho_{2}^{-1}\varpi \frac{t^{\alpha }-t_{0}^{\alpha }}{\alpha }\right). \end{array}$$

Rearranging both sides of the above inequality yields

(15) $$\begin{array}{c} {∥y\left(t\right)∥}^{\mu }\leq \frac{\varrho_{2}}{\varrho_{1}}{∥y\left(t_{0}\right)∥}^{\mu \nu }\exp\left(-\varrho_{3}\varrho_{2}^{-1}\varpi \frac{t^{\alpha }-t_{0}^{\alpha }}{\alpha }\right). \end{array}$$

Let $K={\left(\varrho_{2}/\varrho_{1}\right)}^{1/\mu }$ and $\lambda =\varrho_{3}{\left(\mu \varrho_{2}\right)}^{-1}$. It follows from (15) that

(16) $$\begin{array}{c} ∥y\left(t\right)∥\;\leq \;K∥y\left(t_{0}\right){∥}^{\nu }\exp\left(-\lambda \varpi \frac{t^{\alpha }-t_{0}^{\alpha }}{\alpha }\right). \end{array}$$

Thus, the origin of system (1) is V fractionally exponentially stable. □

**Theorem 2.**  *Suppose y=0 is an equilibrium point of system *(1)* and $L:R_{+}\times R^{n}\to R$ is a continuous function. Moreover, there exists a class K function ϑ such that the following conditions are satisfied:*

*(B1)*  *ϑ(∥y(t)∥)≤L(t,y(t)) and L(t,0)=0;*
*(B2)*  *L(t,y(t)) is V-fractional α-differentiable for all $t>t_{0}\geq 0$;*
*(B3)*  *Vγ,β,αδ,p,qiρL(t,y(t))≤0.*

*Then the origin of system *(1)* is stable.*

**Proof.**  From Lemma 6 and the condition Vγ,β,αδ,p,qiρL(t,y(t))≤0, we have

(17) $$\begin{array}{c} L\left(t,y\left(t\right)\right)\leq L\left(t_{0},y\left(t_{0}\right)\right). \end{array}$$

Combining (B1) and (17) yields $\vartheta \left(∥y\left(t\right)∥\right)\leq L\left(t_{0},y\left(t_{0}\right)\right)$ for all $t\geq t_{0}$.

Fix ε>0. Since $L(t_{0},0)=0$ and *L* is continuous, there exists $\delta =\delta (t_{0},\varepsilon )>0$ such that

(18) $$\begin{array}{c} ∥y_{0}∥\;<\;\delta ⟹L\left(t_{0},y\left(t_{0}\right)\right)<\vartheta \left(\varepsilon \right). \end{array}$$

It follows from (17) and (18) that

(19) $$\begin{array}{c} ∥y_{0}∥\;<\;\delta ⟹\;∥y\left(t\right)∥\;<\;\varepsilon ,\;\forall t\geq t_{0}. \end{array}$$

Thus, the origin of system (1) is stable. This completes the proof of Theorem 2. □

**Theorem 3.**  *Suppose y=0 is an equilibrium point of system *(1)* and $L:R_{+}\times R^{n}\to R$ is a continuous function. Furthermore, there exist three class K functions $\vartheta_{1},\vartheta_{2},\vartheta_{3}$ such that the following conditions hold:*

*(C1)*  *$\vartheta_{1}\left(∥y\left(t\right)∥\right)\leq L\left(t,y\left(t\right)\right)\leq \vartheta_{2}\left(∥y\left(t\right)∥\right)$;*
*(C2)*  *L(t,y(t)) is V-fractional α-differentiable for all $t>t_{0}\geq 0$;*
*(C3)*  *Vγ,β,αδ,p,qiρL(t,y(t))≤−ϑ3(∥y(t)∥).*

*Then the origin of system *(1)* is asymptotically stable. Moreover, if $\vartheta_{1},\vartheta_{2},\vartheta_{3}\in K_{\infty }$, the origin of system *(1)* is globally asymptotically stable.*

**Proof.**  By Theorem 2, the origin of system (1) is stable. Define ${\hat{\vartheta }}_{1}=\lim_{s\to +\infty }\vartheta_{1}\left(s\right)$ and $\tilde{\vartheta }\in \left(0,{\hat{\vartheta }}_{1}\right)$. We choose $y_{0}\in R^{n}$ such that $∥y_{0}∥<\vartheta_{2}^{-1}\left(\tilde{\vartheta }\right)$. Let l(t)=L(t,y(t)). Using condition (C3) and Lemma 6, we conclude that L(t,y(t)) is decreasing. Condition (C1) implies L(t,y(t))≥0, so $\lim_{t\to \infty }l\left(t\right)=l_{0}\geq 0$. Next, we prove that

(20) $$\begin{array}{c} \lim_{t\to \infty }l\left(t\right)=0. \end{array}$$

Use proof by contradiction: suppose $l_{0}>0$. Since L(t,y(t)) decreases, $L\left(t,y\left(t\right)\right)\geq l_{0}>0$ for all $t\geq t_{0}$. According to the condition (C1), we observe that $l_{0}\leq L\left(t,y\left(t\right)\right)\leq L\left(t_{0},y\left(t_{0}\right)\right)\leq \vartheta_{2}(∥y_{0}∥)\leq \tilde{\vartheta }$. Combining the condition (C1) and (C3) yields

(21) Vγ,β,αδ,p,qiρL(t,y(t))≤−ϑ3ϑ2−1(L(t,y(t)))≤−ϑ3ϑ2−1(l0)=−ϑ3ϑ2−1(l0)L(t0,y(t0))L(t0,y(t0))≤−ϑ3ϑ2−1(l0)L(t0,y(t0))L(t,y(t))for∀t>t0.

Let $\lambda_{0}=\vartheta_{3}\left(\vartheta_{2}^{-1}\left(l_{0}\right)\right)/L\left(t_{0},y\left(t_{0}\right)\right)$. The inequality (21) is rewritten as the following form

(22) Vγ,β,αδ,p,qiρL(t,y(t))≤−λ0L(t,y(t))for∀t>t0.

Applying Corollary 1 to the above inequality (22), we acquire

(23) $$\begin{array}{c} L\left(t,y\left(t\right)\right)\leq L\left(t_{0},y\left(t_{0}\right)\right)\exp\left(-\lambda_{0}\varpi \frac{t^{\alpha }-t_{0}^{\alpha }}{\alpha }\right)\;\;\mathrm{for}\;\;\forall t\geq t_{0}, \end{array}$$

which contradicts $L\left(t,y\left(t\right)\right)\geq l_{0}>0$, so (20) holds. Based on the condition (C1), $\lim_{t\to \infty }y\left(t\right)$=0, so the origin of system (1) is attractive. Since stability and attractivity of the origin, it is asymptotically stable.

For $\vartheta_{1},\vartheta_{2},\vartheta_{3}\in K_{\infty }$, along the proof of the first case, we can prove $\lim_{t\to \infty }y\left(t\right)=0$ for all $y_{0}\in R^{n}$. This completes the proof of Theorem 3. □

**Corollary 2.**  *Under the conditions of Theorems 1–3, the conditions (A3), (B3) and (C3) are replaced by ${\left(\partial L\left(t,y\right)/\partial y\right)}^{T}\varphi \left(t,y\right)\leq -\varrho_{3}{∥y\left(t\right)∥}^{\mu \nu }$, ${\left(\partial L\left(t,y\right)/\partial y\right)}^{T}\varphi \left(t,y\right)\leq 0$ and ${\left(\partial L\left(t,y\right)/\partial y\right)}^{T}\varphi \left(t,y\right)$$\leq -\vartheta_{3}\left(∥y\left(t\right)∥\right)$, respectively. Then the conclusions of Theorems 1–3 also hold.*

**Proof.**  It follows from Lemma 2 and the proofs of Theorems 1–3 that we can easily obtain Corollary 2. □

Then, some examples with numerical simulations are presented to verify the effectiveness of the main results.

**Example 1.**  *Consider the truncated V-fractional-order derivative system*

(24) Vγ,β,αδ,p,qiρy1=−2y1+e−ty2,Vγ,β,αδ,p,qiρy2=−52y2+(1+cost)y3,Vγ,β,αδ,p,qiρy3=−3y3+(1+sin(2t))y4,Vγ,β,αδ,p,qiρy4=−52y4+y1,

*where initial values of system *(24)* are $y_{\iota }\left(0.1\right)=y_{\iota 0}$, ι=1,2,3,4. Then the origin of system *(24)* is V fractionally exponentially stable.*

Consider the Lyapunov function candidate $L\left(t,y\left(t\right)\right)={∥y\left(t\right)∥}^{2}/2=\left(y_{1}^{2}+y_{2}^{2}+y_{3}^{2}+y_{4}^{2}\right)/2$. From Remark 4, along the trajectories of system (24), we have

(25) Vγ,β,αδ,p,qiρL(t,y)=y1Vγ,β,αδ,p,qiρy1+y2Vγ,β,αδ,p,qiρy2+y3Vγ,β,αδ,p,qiρy3+y4Vγ,β,αδ,p,qiρy4=y1−2y1+e−ty2+y2−52y2+(1+cost)y3+y3−3y3+(1+sin(2t))y4+y4−52y4+y1=−2y12−52y22−3y32−52y42+e−ty1y2+(1+cost)y2y3+(1+sin(2t))y3y4+y1y4≤−2y12−52y22−3y32−52y42+e−t2(y12+y22)+1+cost2(y22+y32)+1+sin(2t)2(y32+y42)+12(y12+y42)=−2−e−t+12y12−52−e−t+1+cost2y22−3−2+cost+sin(2t)2y32−52−2+sin(2t)2y42≤−(y12+y22+y32+y42)=−∥y(t)∥2.

**Figure 1:**
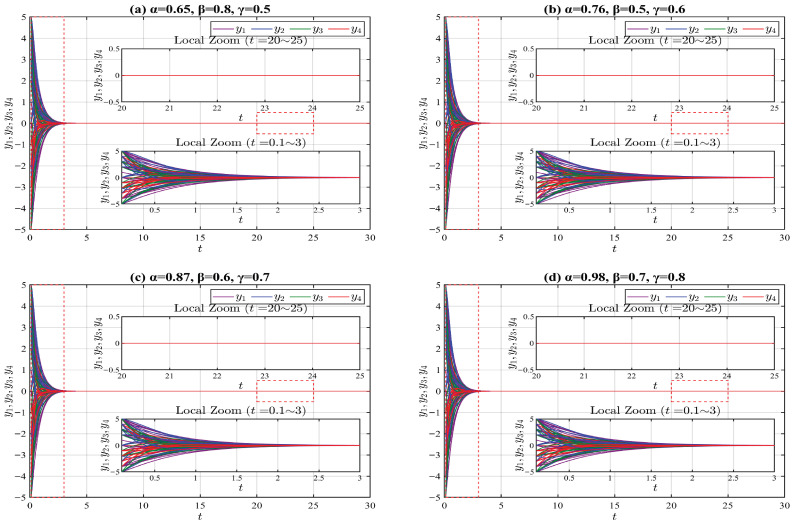
State trajectories of the system (24) for different parameter values. The red dashed boxes mark the magnified portions of the figures.

For system (24), we select 20 sets of initial values near the origin using the following MATLAB R2024a random function: “n_groups = 20; delta = 1; s0_all = −5 + delta*randi([0,10], n_groups)”. We also investigate four types of truncated V-fractional derivatives by setting different parameters: ρ=δ=p=q=0.5, (a) α=0.65, β=0.8, γ=0.5. (b) α=0.76, β=0.5, γ=0.6. (c) α=0.87, β=0.6, γ=0.7. (d) α=0.98, β=0.7, γ=0.8. The numerical simulation results of system (24) are presented in Figure 1.

The conditions of Theorem 1 are satisfied with $\varrho_{1}=\varrho_{2}=1/2$, μ=2, and $\nu =\varrho_{3}=1$. Therefore, the origin of system (24) is V fractionally exponentially stable.

**Example 2.**  *Consider the truncated V-fractional-order derivative system*

(26) Vγ,β,αδ,p,qiρy1=−2y13+e−ty23,Vγ,β,αδ,p,qiρy2=−74y23+(1+cost)y33,Vγ,β,αδ,p,qiρy3=−3y33+(1+sin(2t))y43,Vγ,β,αδ,p,qiρy4=−94y43+y13,

*where initial values of system *(24)* are $y_{\iota }\left(0.1\right)=y_{\iota 0}$, ι=1,2,3,4. Then the origin of system *(26)* is globally asymptotically stable.*

It follows from Young’s inequality: $mn\leq m^{p}/p+n^{q}/q$ for 1/p+1/q=1 with p,q>1. If m=x, $n=y^{3}$, p=4, and q=4/3, then $xy^{3}\leq x^{4}/4+3y^{4}/4$.

Consider the Lyapunov function candidate $L\left(t,y\left(t\right)\right)={∥y\left(t\right)∥}^{2}/2=\left(y_{1}^{2}+y_{2}^{2}+y_{3}^{2}+y_{4}^{2}\right)/2$. From Remark 4, along the trajectories of system (26), we have

(27) Vγ,β,αδ,p,qiρL(t,y)=y1Vγ,β,αδ,p,qiρy1+y2Vγ,β,αδ,p,qiρy2+y3Vγ,β,αδ,p,qiρy3+y4Vγ,β,αδ,p,qiρy4=y1−2y13+e−ty23+y2−74y23+(1+cost)y33+y3−3y33+(1+sin(2t))y43+y4−94y43+y13=−2y14−74y24−3y34−94y44+e−ty1y23+(1+cost)y2y33+(1+sin(2t))y3y43+y4y13≤−2y14−74y24−3y34−94y44+e−ty144+3y244+(1+cost)y244+3y344+(1+sin(2t))y344+3y444+y444+3y144=−2−e−t+34y14−74−3e−t+1+cost4y24−3−4+3cost+sin(2t)4y34−94−4+3sin(2t)4y44≤−(y14+y24+y34+y44)≤−14∥y(t)∥4.

The conditions of Theorem 3 are satisfied with $\vartheta_{1}\left(z\right)=\vartheta_{2}\left(z\right)=z^{2}/2$ and $\vartheta_{3}\left(z\right)=z^{4}/4$. Therefore, the origin of system (26) is globally asymptotically stable.

**Figure 2:**
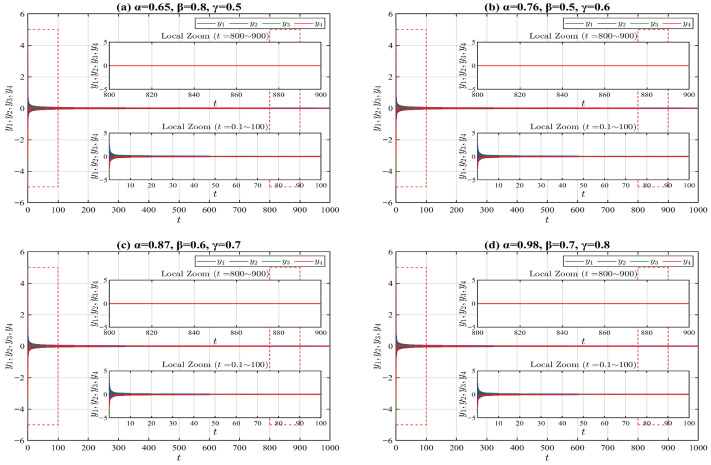
State trajectories of the system (26) for different parameter values. The red dashed boxes mark the magnified portions of the figures.

For system (26), we adopt the identical initial values and truncated V-fractional derivative operators as those utilized in Example 1. The corresponding numerical simulation results of system (26) are depicted in Figure 2. By comparing the numerical simulation outcomes of Examples 1 and 2, a remarkable divergence in convergence speed is clearly identified: the state trajectories of the exponentially stable system corresponding to Example 1, with the horizontal axis ranging over the interval [0,30], converge far more rapidly than those of the asymptotically stable system corresponding to Example 2, with the horizontal axis ranging over the interval [0,1000].

## 4. Applications

In this section, for convenience, system (1) is referred to as the drive system. We introduce the following truncated V-fractional derivative system as the response system:

(28) Vγ,β,αδ,p,qiρx(t)=ψ(t,x(t))+u(t,x(t),y(t)),t>t0,x(t0)=x0,

where $x\in R^{n}$, $\psi :R_{+}\times R^{n}\to R^{n}$ is a given nonlinear function satisfying ψ(t,0)=0, and $u\in R^{n}$ is the adaptive control input.

If there exists a diagonal constant matrix $\sigma =\mathrm{diag}(\sigma_{1},\sigma_{2},\ldots ,\sigma_{n})$ such that $\lim_{t\to \infty }∥x-\sigma y∥=0$, this phenomenon is referred to as modified projective synchronization (MPS), and σ is called the scaling matrix. Clearly, complete synchronization (CS), anti-synchronization (AS) and projective synchronization (PS) are special cases of MPS: CS and AS correspond to $\sigma_{1}=\sigma_{2}=\cdots =\sigma_{n}=1$ and $\sigma_{1}=\sigma_{2}=\cdots =\sigma_{n}=-1$, respectively, while PS corresponds to $\sigma_{1}=\sigma_{2}=\cdots =\sigma_{n}=\sigma_{0}$ (a constant).

Let us define the error e=x−σy corresponding to systems (1) and (28). With this definition, we obtain

(29) Vγ,β,αδ,p,qiρe(t)=Vγ,β,αδ,p,qiρx(t)−σVγ,β,αδ,p,qiρy(t)=ψ(t,x(t))−σφ(t,y(t))+u(t,x(t),y(t))=ϕ(t,e(t),u(t,x(t),y(t))),t>t0,e(t0)=x0−σy0.

**Definition 8.** 

*1.*  *The drive system *(1)* and the response system *(28)* achieve V-fractionally exponential MPS via the feedback control u(t,x(t),y(t)) if the corresponding error system (*29)* is V fractionally exponentially stable.*
*2.*  *The drive system *(1)* and the response system *(28)* achieve asymptotically MPS* (*or globally asymptotically MPS, respectively*)*, via the feedback control u(t,x(t),y(t)) if the associated error system *(29)* is asymptotically stable* (*or globally asymptotically stable, respectively*).

Based on the stability results derived in the previous section, we now present sufficient conditions for the V fractionally exponential MPS and asymptotically MPS of the drive system (1) and the response system (28) via the Lyapunov direct method, and their proofs are omitted.

**Theorem 4.**  *Suppose there exists a continuous function $L:R_{+}\times R^{n}\to R$ satisfying L(t,0)=0 for all t≥0, such that the error system *(29)* admits a Lyapunov function that satisfies conditions (A1)–(A3). Then, the drive system *(1)* and the response system *(28)* are V fractionally exponentially MPS via the feedback control u(t,x(t),y(t)).*

**Theorem 5.**  *Suppose there exists a continuous function $L:R_{+}\times R^{n}\to R$ satisfying L(t,0)=0 for all t≥0, such that the error system *(29)* admits a Lyapunov function satisfying conditions (C1)–(C3), where $\vartheta_{i}\in K$ (or $\vartheta_{i}\in K_{\infty })$ for i=1,2,3. Then, the drive system *(1)* and the response system *(28)* are asymptotically MPS (or globally asymptotically MPS, respectively) via the feedback control u(t,x(t),y(t)). Additionally, if the error system *(29)* satisfies conditions (B1)–(B3), the drive-response systems *(1)*–*(28)* achieve MPS under controller u(t,x(t),y(t)).*

**Figure 3:**
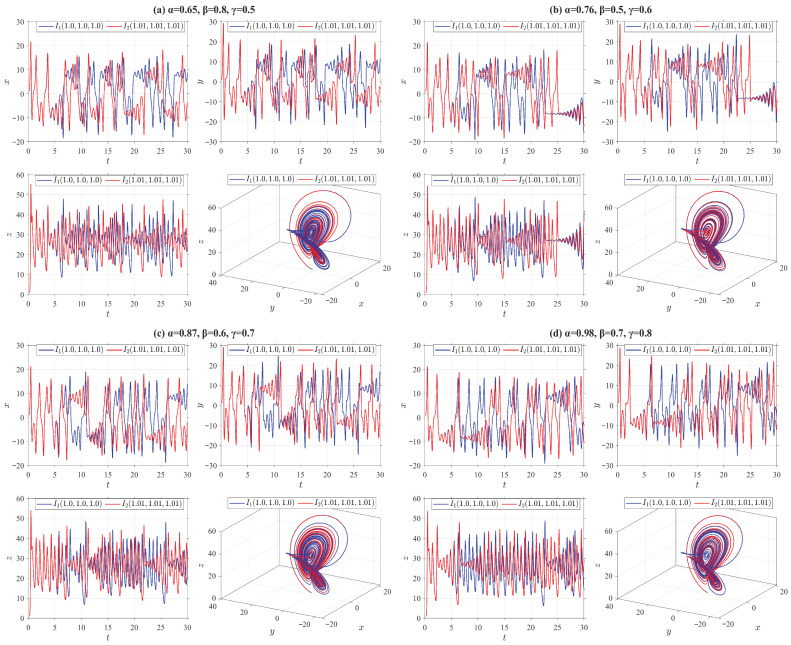
State trajectories of Lorenz system (30) for different parameter values.

**Figure 4:**
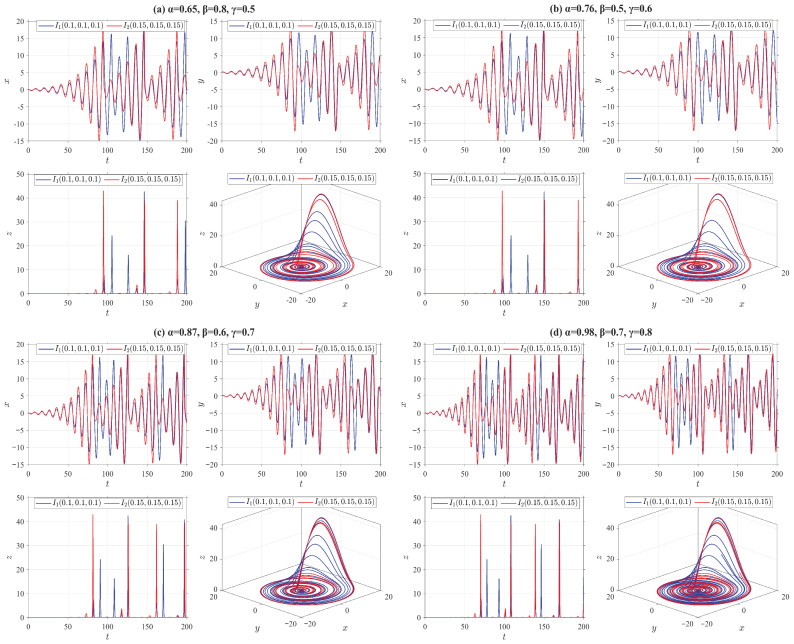
State trajectories of Rössler system (31) for different parameter values.

**Figure 5:**
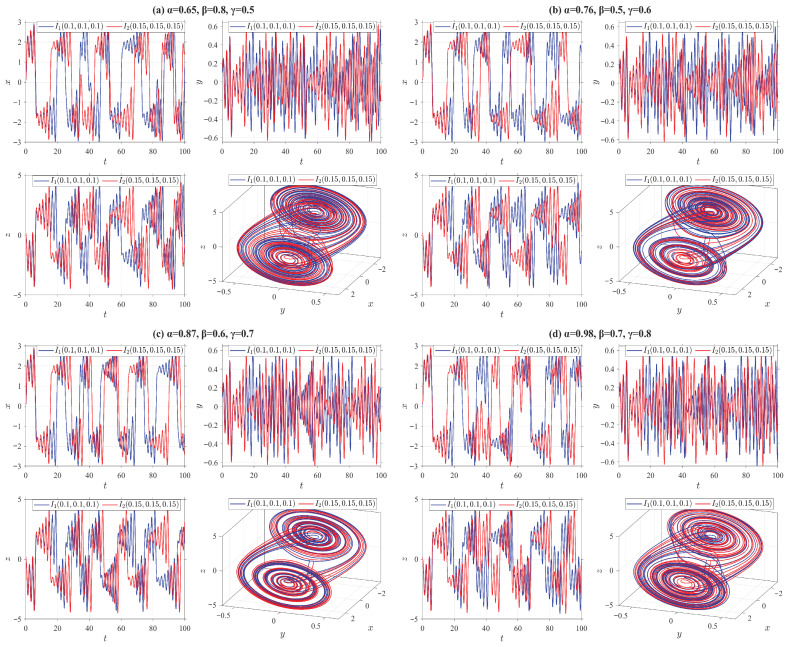
State trajectories of Chua system (32) for different parameter values.

**Figure 6:**
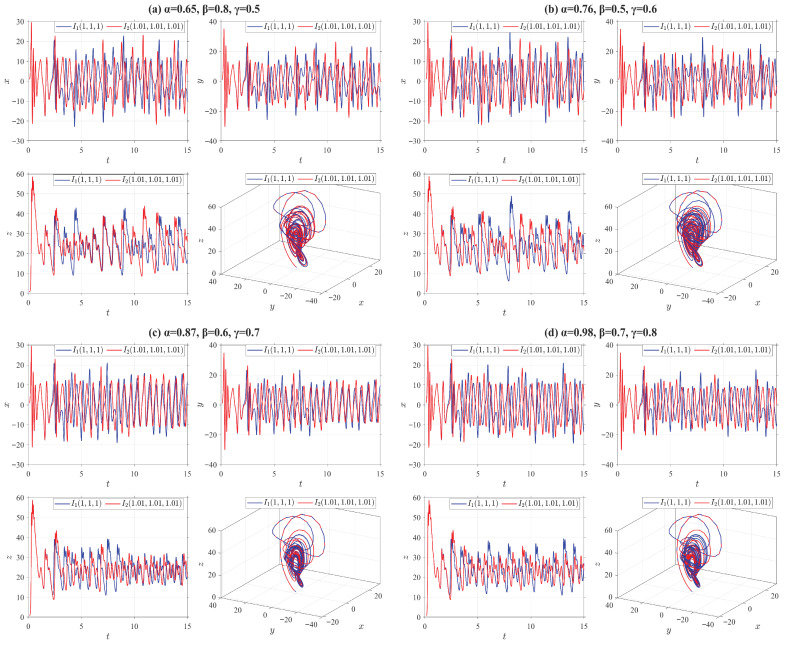
State trajectories of Chen system (33) for different parameter values.

To show the effectiveness of Theorems 4 and 5, we consider several well-known truncated V-fractional derivative chaotic systems.

(D1) The truncated V-fractional derivative Lorenz system
  (30) Vγ,β,αδ,p,qiρx=o1(y−x),Vγ,β,αδ,p,qiρy=x(o2−z)−y,Vγ,β,αδ,p,qiρz=xy−o3z,
  where $o_{1}=10$, $o_{2}=28$, $o_{3}=8/3$. In Figure 3, we present the state trajectories and chaotic characteristics of the truncated V-fractional chaotic system (30) by employing the four categories of truncated V-fractional derivatives from Example 1, along with two groups of initial values $I_{1}=\left(1,1,1\right)$ and $I_{2}=\left(1.01,1.01,1.01\right)$.
(D2) The truncated V-fractional derivative Rössler system
  (31) Vγ,β,αδ,p,qiρx=−o1y−z,Vγ,β,αδ,p,qiρy=o1x+o2y,Vγ,β,αδ,p,qiρz=o3+z(x−o4),
  where $o_{1}=1$, $o_{2}=0.165$, $o_{3}=0.2$, $o_{4}=10$. Figure 4 shows the state trajectories and chaotic behaviors of the truncated V-fractional derivative chaotic system (31) based on the four types of truncated V-fractional derivatives of Example 1, along with two groups of initial values $I_{1}=\left(0.1,0.1,0.1\right)$ and $I_{2}=\left(0.15,0.15,0.15\right)$.
(D3) The truncated V-fractional derivative Chua system
  (32) Vγ,β,αδ,p,qiρx=o1(y−x−f(x)),Vγ,β,αδ,p,qiρy=x−y+z,Vγ,β,αδ,p,qiρz=−o2y,
  where $f\left(x\right)=o_{4}x+0.5\left(o_{3}-o_{4}\right)\left(|x+1|-|x-1|\right)$, $o_{1}=9$, $o_{2}=100/7$, $o_{3}=-1.27$, $o_{4}=-0.68$. In Figure 5, we present the state trajectories and chaotic characteristics of the truncated V-fractional chaotic system (32) by employing the four types of truncated V-fractional derivatives from Example 1 and two sets of initial value configurations: $I_{1}=\left(0.1,0.1,0.1\right)$ and $I_{2}=\left(0.15,0.15,0.15\right)$.
(D4) The truncated V-fractional derivative Chen system
  (33) Vγ,β,αδ,p,qiρx=o1(y−x),Vγ,β,αδ,p,qiρy=(o3−o1)x−xz+o3y,Vγ,β,αδ,p,qiρz=xy−o2z,
  where $o_{1}=35$, $o_{2}=3$, $o_{3}=28$. In Figure 6, the state trajectories and chaotic behaviors of the truncated V-fractional chaotic system (33) are illustrated by using the four types of truncated V-fractional derivatives from Example 1 and two sets of initial value settings: $I_{1}=\left(1,1,1\right)$ and $I_{2}=\left(1.01,1.01,1.01\right)$.

From Figure 3, Figure 4, Figure 5 and Figure 6, it can be observed that all the truncated V-fractional derivative systems exhibit chaotic phenomena even when their key parameters are adjusted within a certain range. Meanwhile, a slight perturbation imposed on the initial values of these systems will immediately induce significant dynamical variations in the chaotic dynamics.

**Figure 7:**
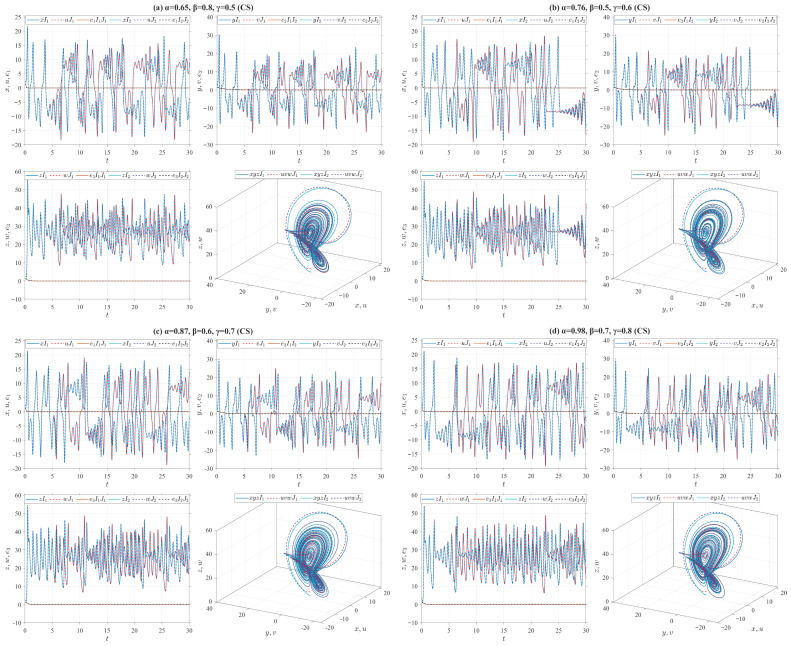
CS between Lorenz systems (30) and (34) for different parameter values.

**Figure 8:**
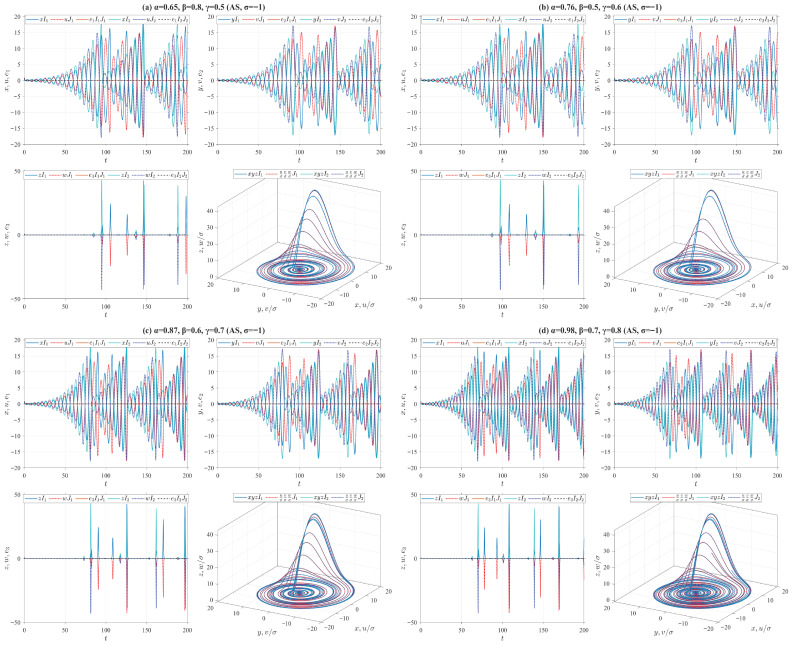
AS between Rössler systems (31) and (35) for different parameter values.

**Figure 9:**
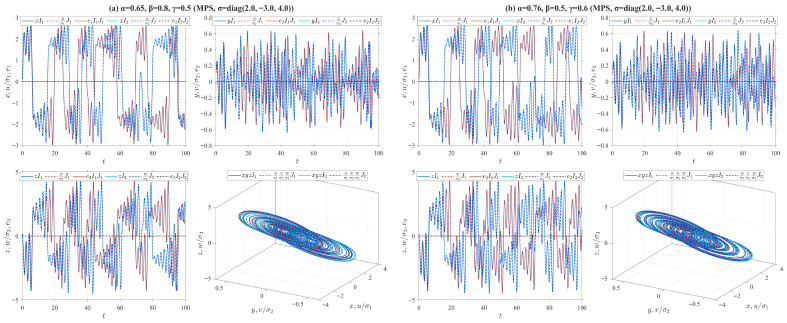

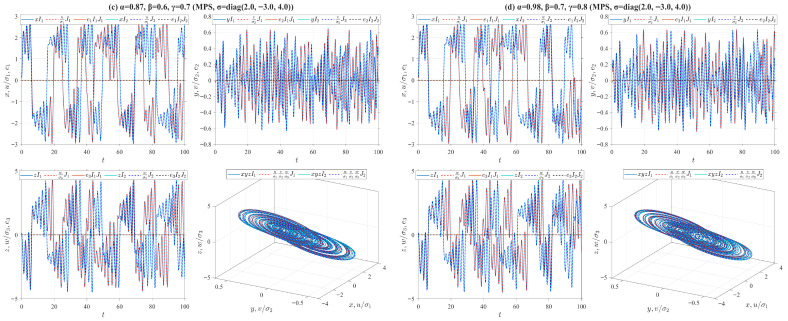
MPS between Chua systems (32) and (36) for different parameter values.

**Figure 10:**
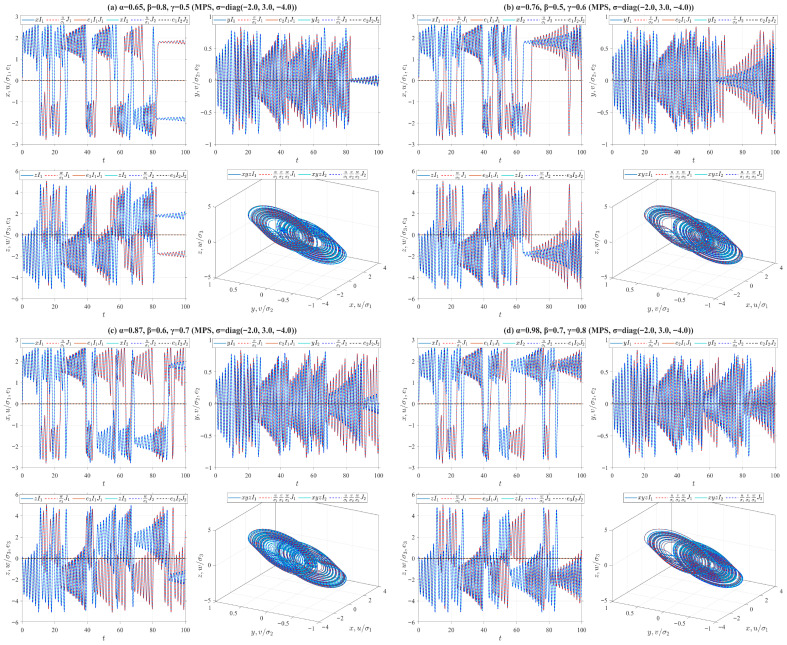
MPS between Chua systems (32) and (36) for different parameter values.

**Figure 11:**
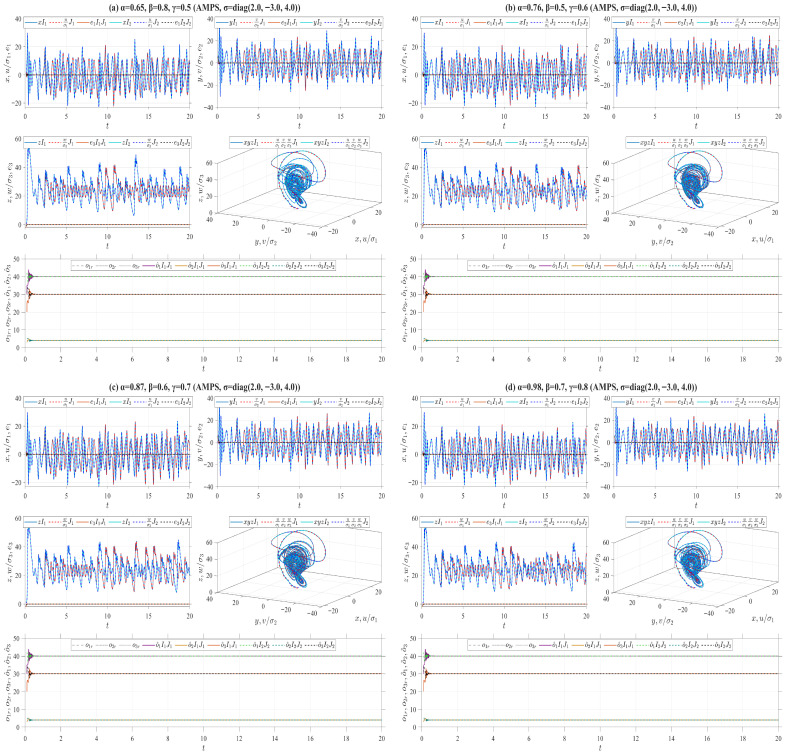
AMPS between Chen systems (33) and (37) for different parameter values.

**Figure 12:**
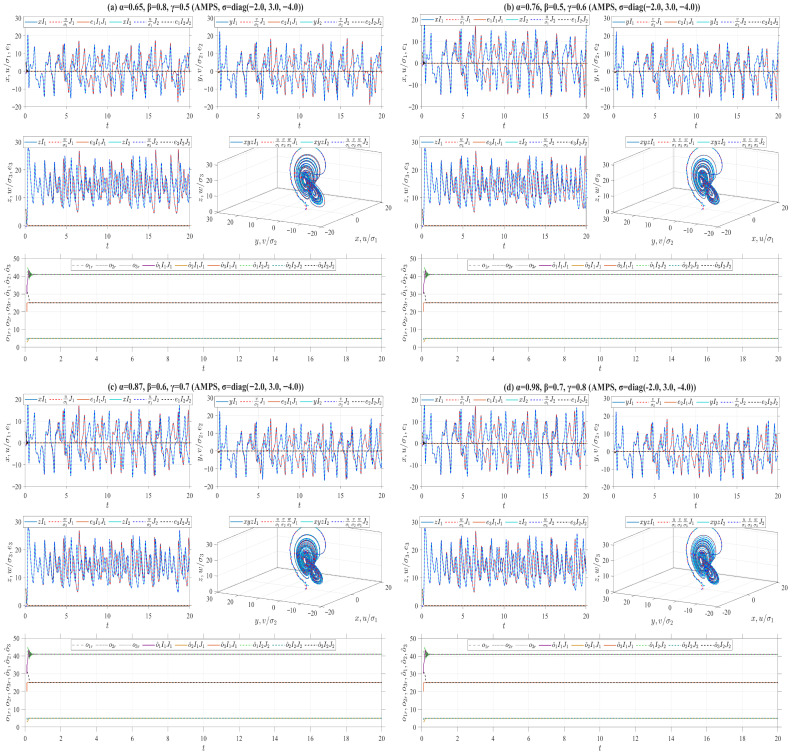
AMPS between Chen systems (33) and (37) for different parameter values.

Next, the synchronization results between the drive and response systems are presented as follows. For clarity, the notations in the legends of the drive-response systems are defined as follows: $xI_{1}$ represents the state trajectory of the state variable *x* with initial condition $I_{1}$; $\frac{u_{1}}{\sigma_{1}}I_{1}$ denotes the state trajectory of the normalized variable $\frac{u_{1}}{\sigma_{1}}$ with initial condition $I_{1}$; $xyzI_{1}$ and $\frac{u}{\sigma_{1}}\frac{v}{\sigma_{2}}\frac{w}{\sigma_{3}}I_{1}$ shows the phase trajectories of the drive system and the response system with initial condition $I_{1}$, respectively; $e_{1}I_{1}J_{1}$ stands for the trajectory of the synchronization error between the drive-response systems corresponding to initial conditions $I_{1}$ and $J_{1}$, respectively. The definitions of other symbols follow the same convention.

(E1) Consider the truncated V-fractional derivative Lorenz system (30) as the drive system and the following Lorenz system as the response system
  (34) Vγ,β,αδ,p,qiρu=o1(v−u)+f1,Vγ,β,αδ,p,qiρv=x(o2−w)−v+f2,Vγ,β,αδ,p,qiρw=uv−o3w+f3,
  where $o_{1}=10$, $o_{2}=28$, $o_{3}=8/3$. Let $e_{1}=u-x$, $e_{2}=v-y$ and $e_{3}=w-z$, then we introduce the control inputs $f_{1}=-o_{1}e_{2}$, $f_{2}=uw-xz-o_{2}e_{1}$ and $f_{3}=xy-uv$. Define the Lyapunov function $V\left(t\right)=\left(e_{1}^{2}+e_{2}^{2}+e_{3}^{2}\right)/2$. According to Remark 4, we obtain Vγ,β,αδ,p,qiρV(t)=−o1e12−e22−o3e32≤−o3(e12+e22+e32)=−2o3V(t). According to Theorem 1, the origin of the error system between the drive system (30) and the response system (34) is V fractionally exponentially stable. Furthermore, Theorem 4 ensures the achievement of V-fractionally exponential CS between these two systems. For numerical simulations, the drive system (30) is initialized with two sets of initial values: $I_{1}=\left(1,1,1\right)$ and $I_{2}=\left(1.01,1.01,1.01\right)$, while the response system (34) adopts $J_{1}=\left(2.5,2.5,2.5\right)$ and $J_{2}=\left(2.51,2.51,2.51\right)$ as its initial conditions. Figure 7 presents numerical simulations of the CS state trajectories and chaotic characteristics between the drive system (30) and the response system (34), utilizing the four types of truncated V-fractional derivatives introduced in Example 1.
(E2) Consider the truncated V-fractional derivative Rössler system (31) as the drive system and the following Rössler system as the response system
  (35) Vγ,β,αδ,p,qiρu=−o1v−w+f1,Vγ,β,αδ,p,qiρv=o1u+o2v+f2,Vγ,β,αδ,p,qiρw=o3+w(u−o4)+f3,
  where $o_{1}=1$, $o_{2}=0.165$, $o_{3}=0.2$, $o_{4}=10$. Let $e_{1}=u+x$, $e_{2}=v+y$ and $e_{3}=w+z$, then we introduce the control inputs $f_{1}=o_{1}e_{2}+e_{3}-e_{1}$, $f_{2}=-o_{1}e_{1}-\left(o_{2}+1\right)e_{2}$ and $f_{3}=-2o_{3}-xy-uv$. Define the Lyapunov function $V\left(t\right)=\left(e_{1}^{2}+e_{2}^{2}+e_{3}^{2}\right)/2$. According to Remark 4, we obtain Vγ,β,αδ,p,qiρV(t)=−e12−e22−o3e32≤−o3(e12+e22+e32)=−2o3V(t). It follows from Theorem 1 that the origin of the error system between the drive system (31) and the response system (35) is V-fractionally exponentially stable. Furthermore, from Theorem 4, the drive system (31) and the response system (35) achieve V-fractionally exponential AS. In numerical simulations, two sets of initial values for the drive system (31) are $I_{1}=\left(0.1,0.1,0.1\right)$ and $I_{2}=\left(0.15,0.15,0.15\right)$, while those for the response system (35) are $J_{1}=\left(0.45,0.45,0.45\right)$ and $J_{2}=\left(0.65,0.65,0.65\right)$. In Figure 8, by employing the four types of truncated V-fractional derivatives from Example 1, we numerically simulate the state trajectories of AS between the drive system (31) and the response system (35) and their chaotic characteristics.
(E3) Consider the truncated V-fractional derivative Chua system (32) as the drive system and the following Chua system as the response system
  (36) Vγ,β,αδ,p,qiρu=o1(v−u−f(u))+f1,Vγ,β,αδ,p,qiρv=u−v+w+f2,Vγ,β,αδ,p,qiρw=−o2v+f3,
  where $f\left(u\right)=o_{4}u+0.5\left(o_{3}-o_{4}\right)\left(|u+1|-|u-1|\right)$, $o_{1}=9$, $o_{2}=100/7$, $o_{3}=-1.27$, $o_{4}=-0.68$. Let $e_{1}=u-\sigma_{1}x$, $e_{2}=v-\sigma_{2}y$ and $e_{3}=w-\sigma_{3}z$, then we introduce the control inputs $f_{1}=-ke_{1}-o_{1}\left(v-f\left(u\right)-\sigma_{1}\left(y-f\left(x\right)\right)\right)$, $f_{2}=-ke_{2}-e_{1}-e_{3}-\left(\sigma_{1}-\sigma_{2}\right)x-\left(\sigma_{3}-\sigma_{2}\right)z$ and $f_{3}=-ke_{3}+o_{2}e_{2}-o_{2}\left(\sigma_{3}-\sigma_{2}\right)y$, where *k* is the control gain of the drive-response system. Define the Lyapunov function $V\left(t\right)=\left(e_{1}^{2}+e_{2}^{2}+e_{3}^{2}\right)/2$. According to Remark 4, we obtain Vγ,β,αδ,p,qiρV(t)=−(k+o1)e12−ke22−ke32≤−k(e12+e22+e32)=−2kV(t). It follows from Theorem 1 that the origin of the error system between the drive system (32) and the response system (36) is V-fractionally exponentially stable. Furthermore, from Theorem 4, the drive system (32) and the response system (36) realize V-fractionally exponential MPS. In numerical simulations, we set k=20. Two sets of initial values for the drive system (32) are $I_{1}=\left(0.1,0.1,0.1\right)$ and $I_{2}=\left(0.11\sigma_{1},0.11\sigma_{2},0.11\sigma_{3}\right)$, while those for the response system (36) are $J_{1}=\left(0.15,0.15,0.15\right)$ and $J_{2}=\left(0.16\sigma_{1},0.16\sigma_{2},0.16\sigma_{3}\right)$, where σ=(2,−3,4). In Figure 9, by employing the four types of truncated V-fractional derivatives from Example 1, we numerically simulate the state trajectories of MPS between the drive system (32) and the response system (36) and their chaotic characteristics.
  On the other hand, the following parameter substitutions are made in the drive-response systems (32)–(36): $o=\left(o_{1},o_{2},o_{3},o_{4}\right)=\left(9,100/7,-1.27,-0.68\right)$ is replaced with o=(11,100/7,−11/7,−2/7), and σ=(2,−3,4) with σ=(−2,3,−4). Accordingly, we conduct numerical simulations on the MPS state trajectories and corresponding chaotic behaviors between the drive system (32) and the response system (36) in Figure 10. It follows from Figure 9 and Figure 10 that both the system parameters and the fractional order have a considerable effect on the simulation results.
(E4) Consider the truncated V-fractional derivative Chen system (33) as the drive system and the following Chen system as the response system
  (37) Vγ,β,αδ,p,qiρu=o1(v−u)+f1,Vγ,β,αδ,p,qiρv=(o3−o1)u−uw+o3v+f2,Vγ,β,αδ,p,qiρw=uv−o2w+f3,
  where $o_{1},o_{2},o_{3}$ are three unknown parameters and $o_{1r},o_{2r},o_{3r}$ denote the actual values of unknown parameters $o_{1},o_{2},o_{3}$, respectively. Let $e_{1}=u-\sigma_{1}x$, $e_{2}=v-\sigma_{2}y$ and $e_{3}=w-\sigma_{3}z$, then we introduce the control inputs $f_{1}=-ke_{1}+\sigma_{1}o_{1}\left(y-x\right)-{\hat{o}}_{1}\left(v-u\right)$, $f_{2}=-ke_{2}+\sigma_{2}\left(\left(o_{3}-o_{1}\right)x-xz+o_{3}y\right)-\left(\left({\hat{o}}_{3}-{\hat{o}}_{1}\right)u-uw+{\hat{o}}_{3}v\right)$ and $f_{3}=-ke_{3}+\sigma_{3}\left(xy-o_{2}z\right)-\left(uv-{\hat{o}}_{2}w\right)$, where *k* is a positive parameter, and ${\hat{o}}_{1},{\hat{o}}_{2},{\hat{o}}_{3}$ denote the estimated values of unknown parameters $o_{1},o_{2},o_{3}$, respectively. Define the estimated errors ${\tilde{o}}_{\iota }={\hat{o}}_{\iota }-o_{\iota }$, ι=1,2,3. Next, we design the adaptive laws for the unknown parameters as follows
  (38) Vγ,β,αδ,p,qiρo^1=ℏ1(e1(v−u)−e2u),Vγ,β,αδ,p,qiρo^2=−ℏ2e3w,Vγ,β,αδ,p,qiρo^3=ℏ3e2(u+v),
  where $\hbar_{1},\hbar_{2},\hbar_{3}$ are three designed positive parameters. Define the Lyapunov function $V\left(t\right)=V_{1}\left(t\right)+V_{2}\left(t\right)$, where $V_{1}\left(t\right)=\left(e_{1}^{2}+e_{2}^{2}+e_{3}^{2}\right)/2$ and $V_{2}\left(t\right)={\tilde{o}}_{1}^{2}/\left(2\hbar_{1}\right)+{\tilde{o}}_{2}^{2}/\left(2\hbar_{2}\right)+{\tilde{o}}_{3}^{2}/\left(2\hbar_{3}\right)$. According to Remark 4, we derive Vγ,β,αδ,p,qiρV(t)=−k(e12+e22+e32)≤0. Theorem 2 further implies that under the effect of the adaptive laws (38), the origin of the error system between the drive system (33) and the response system (37) is stable. Moreover, the adaptive MPS (AMPS) is achieved between these two systems by virtue of Theorem 5. For numerical simulations, let $o_{r}=\left(o_{1r},o_{2r},o_{3r}\right)=\left(40,4,30\right)$, ℏ=(7,7,7), k=15 and σ=(2,−3,4). The drive system (33) is initialized with two sets of initial values: $I_{1}=\left(1,1,1\right)$ and $I_{2}=\left(2,2,2\right)$. Correspondingly, the response system (37) adopts $J_{1}=\left(1.01,1.01,1.01\right)$ and $J_{2}=\left(3.01,3.01,3.01\right)$ as its initial conditions, while the initial estimates of the unknown parameters in the adaptive law are ${\hat{o}}_{0,1}=\left(30,3,20\right)$ and ${\hat{o}}_{0,2}=\left(42,5,31\right)$, respectively. Utilizing the four types of truncated V-fractional derivatives introduced in Example 1, Figure 11 presents numerical simulations of the MPS state trajectories and their chaotic behaviors between the drive system (33) and the response system (37).
  On the other hand, the following parameter substitutions are made in the drive-response systems (33)–(37): $o=\left(o_{1},o_{2},o_{3}\right)=\left(35,3,28\right)$ is replaced with o=(42,3,28), σ=(2,−3,4) with σ=(−2,3,−4), $o_{r}=\left(40,4,30\right)$ with $o_{r}=\left(41,5,25\right)$, respectively. Accordingly, we conduct numerical simulations on the MPS state trajectories and corresponding chaotic behaviors between the drive system (33) and the response system (37) in Figure 12. It follows from Figure 11 and Figure 12 that as the drive-response systems (33)–(37) tend to synchronization, the unknown parameters in the response system (37) converge to the predefined true values.

**Figure 13:**
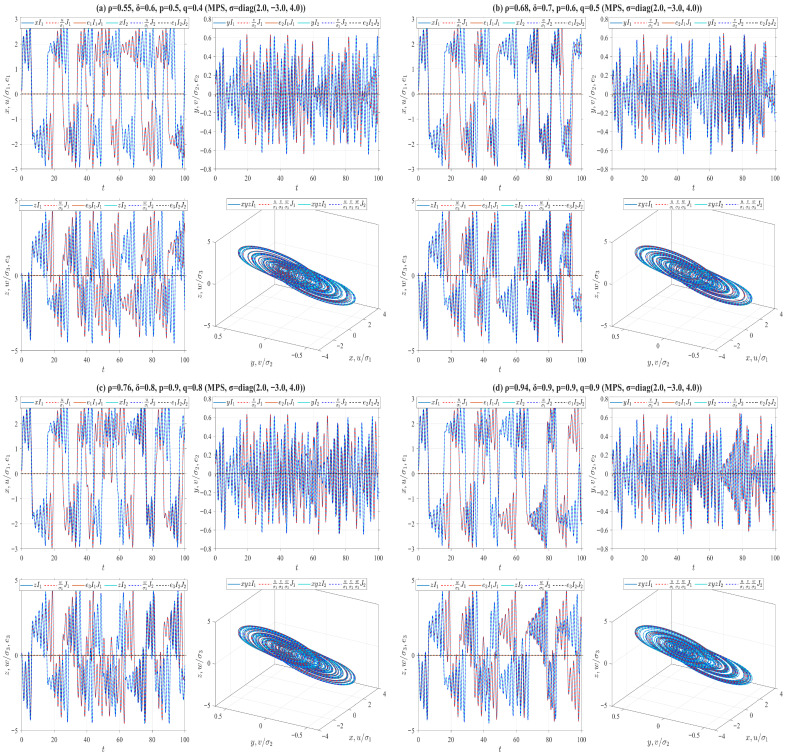
MPS between Chua systems (32) and (36) with fixed parameters α=0.98, β=0.7, γ=0.8 and different parameters ρ,δ,p,q.

**Figure 14:**
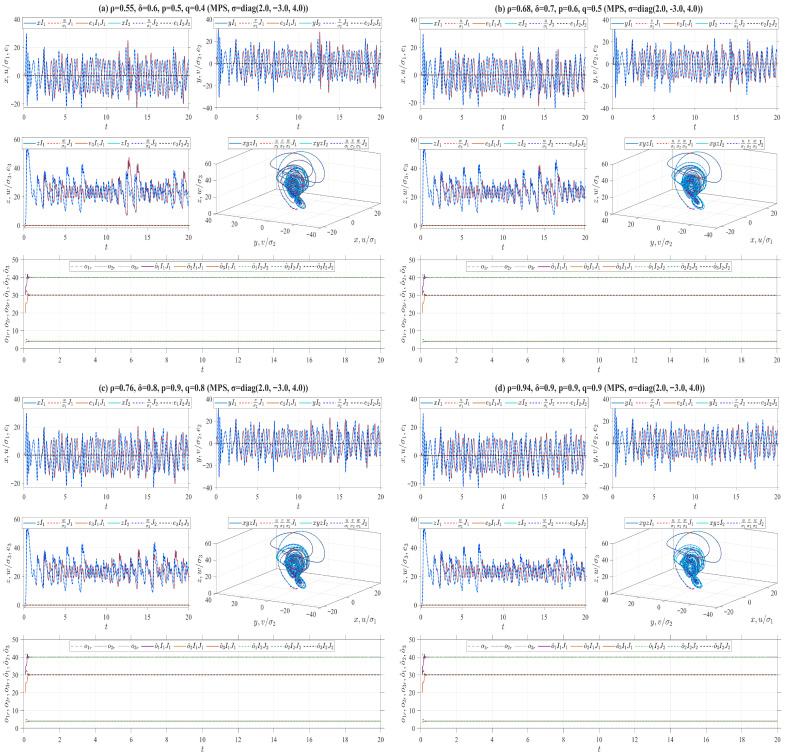
AMPS between Chen systems (33) and (37) with fixed parameters α=0.48, β=0.3, γ=0.4 and different parameters ρ,δ,p,q.

**Figure 15:**
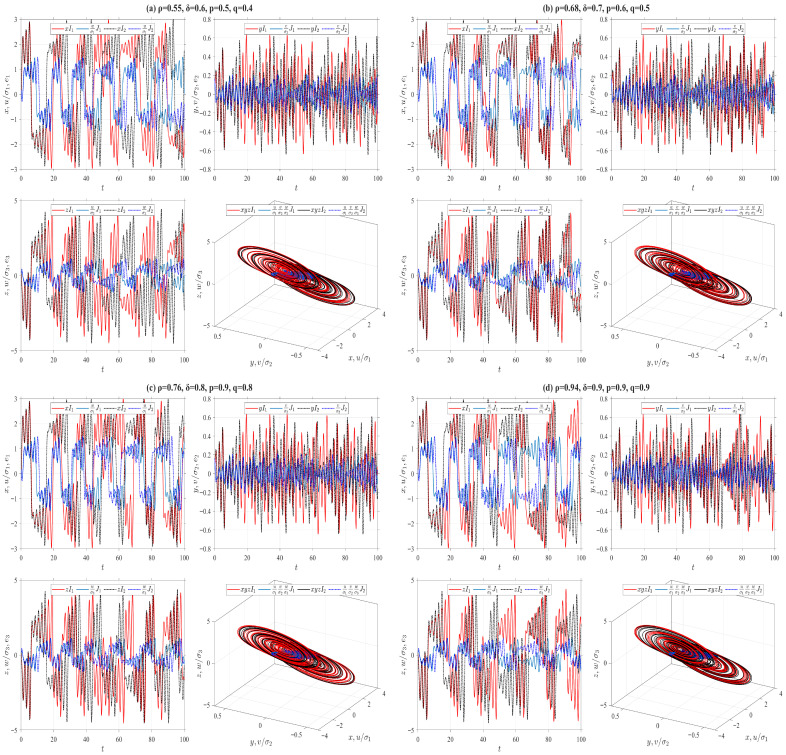
Uncontrolled asynchronous state trajectories between Chua systems (32) and (36) with fixed parameters α=0.98, β=0.7, γ=0.8 and different parameters ρ,δ,p,q.

In the preceding numerical simulations, we set ρ=δ=p=q=0.5 for the truncated V-fractional derivatives, and analyze the synchronization trajectories of the drive-response systems by varying the values of α,β,γ, as illustrated in Figure 6, Figure 7, Figure 8, Figure 9, Figure 10, Figure 11 and Figure 12. The conformable fractional derivative is governed solely by the parameter α, whereas the truncated *M*-fractional derivative is only associated with α and γ. Accordingly, analogous synchronization results can be derived for the drive-response systems involving the conformable fractional derivative and the truncated *M*-fractional derivative. To demonstrate the influence of other parameters in the truncated V-fractional derivative on the system dynamics, we fix the parameters α,β,γ and explore the synchronization trajectories of the aforementioned drive-response systems by tuning the parameters ρ,δ,p,q. Specifically, four sets of parameter values are selected as follows: (a) ρ=0.55, δ=0.6, p=0.5, q=0.4; (b) ρ=0.68, δ=0.7, p=0.6, q=0.5; (c) ρ=0.76, δ=0.8, p=0.9, q=0.8; (d) ρ=0.94, δ=0.9, p=0.9, q=0.9. With the proposed controller activated, Figure 13 and Figure 14 illustrate the synchronized dynamics corresponding to the first scenarios of drive-response systems (E3) (α=0.98, β=0.7, γ=0.8) and drive-response systems (E4) (α=0.48, β=0.3, γ=0.4). In contrast, under identical parameters and without control efforts, Figure 15 verifies that drive-response systems (E3) fails to achieve synchronization.

From the foregoing numerical simulations, it can be observed that every parameter associated with the truncated V-fractional derivative has a notable influence on the overall dynamics of the system. Therefore, the truncated V-fractional derivative systems can better characterize complex dynamical scenarios.

## 5. Conclusions

This paper investigates the stability of nonlinear truncated V-fractional-order derivative systems. The key findings are summarized as follows: First, based on the fundamentals of V-fractional calculus, the Bellman–Gronwall inequality for V-fractional α-differentiable functions is derived, which lays a solid theoretical foundation for the stability analysis of such systems. Second, several sufficient conditions for the stability of the considered systems are established via the Lyapunov direct method, which enriches the stability theory of fractional-order systems. Furthermore, by applying these stability results, multiple synchronization criteria for drive-response systems are deduced, extending the theoretical framework to the field of synchronization control for truncated V-fractional-order systems. Finally, numerical examples are provided to verify the effectiveness and feasibility of the main results, which provides strong support for the theoretical research of truncated V-fractional-order systems. Based on the research findings of this paper, future work will focus on the adaptive control problem of uncertain truncated V-fractional-order derivative systems, aiming to further expand the application scope of the proposed theory.

## Data Availability

Data is contained within the article.
